# Role of Screening Lung Function Tests in a Routine Health Checkup

**DOI:** 10.7759/cureus.49430

**Published:** 2023-11-26

**Authors:** Shankar Iyer, S P Rai, Sumeet Singhania, Caroline Simon

**Affiliations:** 1 Respiratory Medicine, Kokilaben Dhirubhai Ambani Hospital and Medical Research Institute, Mumbai, IND

**Keywords:** health checkup, obstructive airway diseases, air flow obstruction, smoking, "spirometry", lung function test

## Abstract

Background and objectives

The lung function test is a gold standard, guideline-recommended test to detect obstructive airway diseases like asthma and COPD. It is of considerable value in detecting the presence and severity of airflow obstruction in patients with respiratory symptoms. However, the role of spirometry in a routine health checkup is controversial. Spirometry, when used in routine health checkup settings as a case-finding tool for all adults with persistent respiratory symptoms or having a history of exposure to risk factors, is likely to label a relatively large proportion of individuals as diseased with airflow obstruction. Conversely, spirometry is normal in a relatively large percentage of adults who report respiratory symptoms including dyspnea, the respiratory symptom having the greatest impact on quality of life. The objective of this study is to determine the utility of spirometry as a screening test to detect airflow obstruction in otherwise healthy subjects undergoing a routine health checkup.

Methods

This observational study was conducted with 538 health checkup individuals aged 18 and over. A brief history was taken prior to the test. Lung function tests were performed and interpreted as per the Global Initiative for Chronic Obstructive Lung Disease criteria. The anthropometric and spirometric data obtained were compared to other population-based spirometric studies to compare the prevalence of airflow limitation, the risk factors, and smoking history.

Results

Of the total 538 subjects incorporated in the study, 305 (57%) were males and 233 (43%) were females aged between 18 to 80 years with a mean age of 45 years. The male-to-female ratio was 1.3:1 with a mean BMI of 25.9. The overall yield from lung function tests in detecting airflow obstruction was 63 subjects (11.7%), of which 36 (11.8%) were males and 27 (11.5%) were females. Seventy-three subjects (13.5%) were classified as having a small airway obstruction, of which 34 were males (46.6%), and 39 were females (53.4%). The distribution of airflow obstruction by age was with eight subjects (5.4%) in the 18-35 group, 21 subjects (7.8%) in the 36-55 group, and 34 (25%) in the elderly (>55) age group. Although overall smoking history showed no significant association with developing airflow obstruction, significant association with smoking was found in the elderly (>55) age group.

Interpretation and conclusions

The results of the study suggest that lung function tests should be included in routine health checkups in the subset of individuals greater than 35 years of age with or without a history of smoking, in all age groups with a family history of asthma, in individuals with respiratory symptoms and in individuals greater than 55 years of age with a moderate history of smoking.

## Introduction

Spirometry is a physiological test that measures how an individual inhales or exhales volumes of air as a function of time. It is invaluable as a screening test for general functional respiratory health. Spirometry is the gold-standard, guideline-recommended test for the diagnosis of obstructive airway diseases (OADs), including asthma and chronic obstructive pulmonary disease (COPD) [1]. It also helps distinguish between the two diseases, offers a useful index of severity and prognosis, and helps guide appropriate pharmacotherapy [2]. Spirometry is a readily available, safe, cost-effective, and noninvasive test to measure the functional capacity of the lungs.

The most important aspects of spirometry are the forced vital capacity (FVC), which is the volume of air delivered during an expiration made as forcefully and completely as possible starting from full inspiration, and the forced expiratory volume in one second (FEV_1_), which is the volume delivered in the first second of the FVC maneuver. Spirometry is accepted as the diagnostic test to assess airflow obstruction and classify the severity of disease, based on specific cut points: FEV_1_/FVC ratio less than 0.7 and FEV_1_ (mild ≥80% predicted, moderate 50-80%, severe 30-50% predicted, and very severe <30% predicted) [3].

However, in spite of spirometric standards for diagnosis, a high proportion of airway disease in the community remains undiagnosed. Smoking is the most common cause of chronic respiratory illnesses. The prevalence of smoking is increasing overall, particularly in the young population. Air pollution is rapidly becoming a global health problem and is increasingly contributing to respiratory comorbidities. Cumulative exposure to air pollution is a risk factor for developing lung disease. Spirometric screening may help identify such a population earlier. Vehicular traffic and industrial and commercial activities contribute to urban air pollution, whereas biomass fuel use in villages contributes to rural air pollution. Patients at an early stage of the disease either are unaware of their condition or reluctant to consult for their respiratory symptoms. The common occurrence of undiagnosed airflow obstruction in general practices and population-based surveys suggests a need for widespread use of spirometry for early detection.

On the other hand, routine screening spirometric testing in a health checkup setting is likely to result in considerable testing and treatment costs, resource utilization, and healthcare personnel time. It might reduce the number of individuals being labeled as having COPD or receiving disease-specific treatment in the absence of severe to very severe airflow obstruction. However, it is likely to label a large number of individuals who may not be reporting bothersome respiratory symptoms as diseased and who would not benefit from testing or treatment [4].

Mumbai, which is regarded as the financial capital of India, is a melting pot because of migrants from other states that relocate to the city for employment opportunities. Urban air pollution is also prevalent in Mumbai because of industrial, commercial, and vehicular emissions. Because of these reasons, a sample population can be considered representative of the general urban population.

Through our study, we attempt to establish the utility of spirometry as a primary screening test for respiratory diseases in the general population. We aim to determine whether it should be included as a part of a routine health checkup in all subjects undergoing a routine health checkup or as a screening test only in a certain subset of the population. This can help us establish a target population for screening spirometry, which can help us diagnose respiratory problems earlier and provide definitive interventions at an earlier stage. This will also help decrease the unnecessary use of spirometry, thereby reducing treatment costs and saving healthcare personnel time.

## Materials and methods

This observational study was conducted within the outpatient division of the Pulmonary Medicine department at Kokilaben Dhirubhai Ambani Hospital and Medical Research Institute. Prior to commencement, the study received approval from the Institutional Study Ethics Board (ISEB approval number C-3/13-17). The research included individuals of all genders aged 18 and above who were undergoing routine health checkups and had signed a consent form. Participants who had previous spirometry diagnosed obstructive or restrictive airway defects, and those who were unable to perform the spirometry maneuver were excluded from the study.

On the basis of the literature [5], it was found that the detection rates of obstructive airway disease were 4% in the population. For our sample size calculation, considering detection rates of obstructive airway disease to be 4% in the population, with a 5% allowable variation, the sample size at 95% confidence level was calculated to enroll 60 positive patients in this study.

The sample size (n) was calculated according to the formula: n = [z^2^ * p * (1 - p)] / e^2^], where: z = 1.96 for a confidence level (α) of 95%, p = proportion (expressed as a decimal), and e = margin of error.

All the participants were asked to fill up a questionnaire prior to the test (Appendix 1). Age, sex, weight, height, smoking history, occupation, respiratory symptoms, and family history of respiratory illnesses were recorded.

Spirometry was performed using a spirometer device in strict accordance with the American Thoracic Society (ATS) quality control standards. Values like forced vital capacity (FVC), forced expiratory volume in the first second (FEV_1_), FEV_1_/FVC ratio, and forced expiratory flow in mid-expiration (FEF_25% - 75%_) were acquired. Each test was repeated a minimum of three times and a maximum of five times with adequate rest after each maneuver for best results. All the staff involved in lung function tests are qualified professionals who have received special training.

All the results were interpreted for the presence of obstructive airway disease according to the Global Initiative for Chronic Obstructive Lung Disease criteria of FEV_1_/FVC ratio of less than 70%. The obstructive airway diseases were further classified on the basis of FEV_1_% values as per Table 1.

**Table 1:** Severity grading of obstructive airway disease. FEV_1_% - Forced expiratory volume in the first second of forced expiration expressed in percentage.

Severity	FEV_1_%
Mild	>80%
Moderate	50-80%
Severe	30-50%
Very severe	<30%

Small airway disease was diagnosed according to maximal mid-expiratory flow (FEF _25% -75%_) below 60% of predicted in the presence of a normal FEV_1_/FVC. Restrictive airway disease was interpreted as an FEV_1_/FVC ratio greater than 70%, with FVC of less than 70% of predicted.

Statistical analysis

Numeric data are summarized by descriptive statistics like n, MeanSD, median, minimum, and maximum. The categorical data were summarized by frequency count, and percentage and significance were analyzed using a chi-square test. The level of significance was fixed at p=0.05, and any value less than or equal to 0.05 was considered statistically significant. Student t-tests (two tailed, unpaired) were used to find the significance of study parameters on a continuous scale between two groups. An analysis of variance (ANOVA) was used to find the significance of study parameters between the groups (intergroup analysis). The statistical software IBM SPSS Statistics 20.0 (IBM SPSS Statistics for Windows, Armonk, NY) was used for the analyses of the data, and Microsoft Word and Excel were used to generate graphs, tables, and so on.

## Results

A total of 538 subjects aged between 18 and 80 based on the inclusion criteria were incorporated into the study. There were 305 males and 233 females with a male-to-female ratio of 1.3:1. Table 2 shows the baseline parameters of the study subjects and also the mean FEV_1_ across each age group and sex.

**Table 2:** Baseline characteristics of subjects of each age group and sex. FEV_1_% - Forced expiratory volume in the first second of forced expiration expressed in percentage. BMI - Body mass index.

Variables	Sex	Age groups (mean + SD)			Total
		<35 Years	36-55 Years	>55 Years	
N (%)	Males	68 (45%)	144 (57%)	93 (68%)	305 (57%)
	Females	80 (55%)	110 (43%)	43 (32%)	233 (43%)
	Total	148	254	136	538
BMI (kg/m^2^)	Males	24.9 ± 3.7	26.1 ± 3.8	26.6 ± 3.9	26.0 ± 3.9
	Females	23.2 ± 5.5	26.9 ± 4.9	27.4 ± 5.2	25.7 ± 5.4
FEV_1_%	Males	79.7 ± 9	79.7 ± 8.6	81.5 ± 8	80.3 ± 8.7
	Females	81.5 ± 6	80.4 ± 7.3	79.2 ± 8.8	80.5 ± 7.3

**Figure 1:**
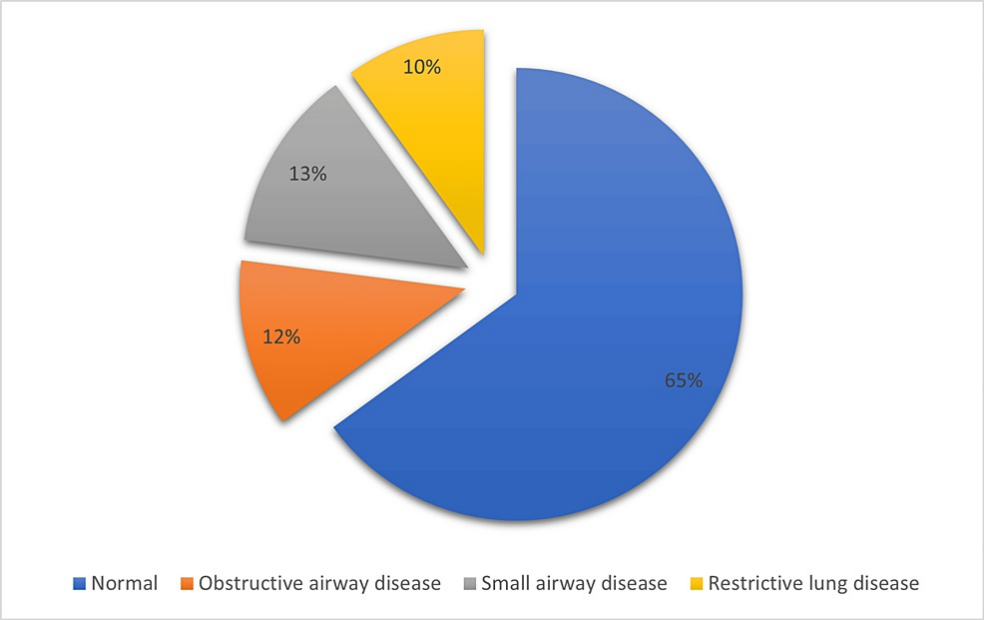
Pie chart showing a distribution of spirometric findings.

Spirometry results were interpreted as per GOLD guidelines. Overall, 347 subjects (64.4%) had normal spirometry, 63 subjects (11.7%) had obstructive defects, 73 subjects (13.5%) were classified as having small airway disease, and 55 individuals (10.2%) demonstrated restrictive defects (Figure 1).

**Figure 2:**
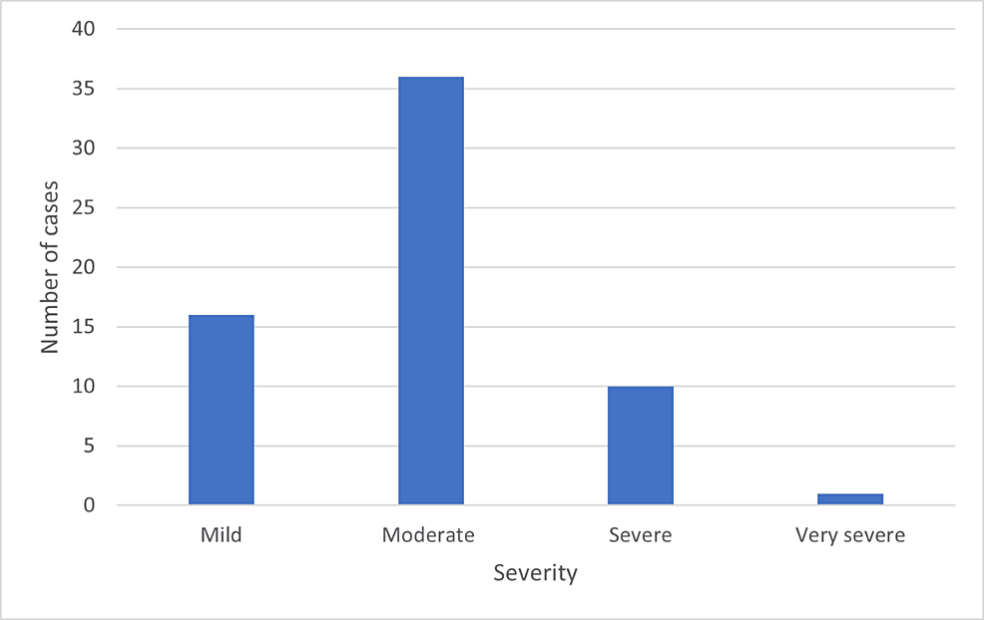
Obstructive lung disease according to severity.

Of the 63 subjects who had obstructive spirometry, 16 subjects (25.4%) had a mild degree of obstructive defect, 36 (57.2%) subjects had a moderate obstructive defect, 10 (15.9%) had a severe obstructive defect, and only one (1.5%) spirometry demonstrated very severe obstructive defect (Figure 2). Thirty-six males (11.8%) out of total 305 total males and 27 females (11.5%) out of 233 total females were diagnosed with obstructive patterns on spirometry according to GOLD guidelines. Of the 73 subjects who were classified as having small airway obstruction, 34 were males (46.6%), and 39 were females (53.4%).

**Figure 3:**
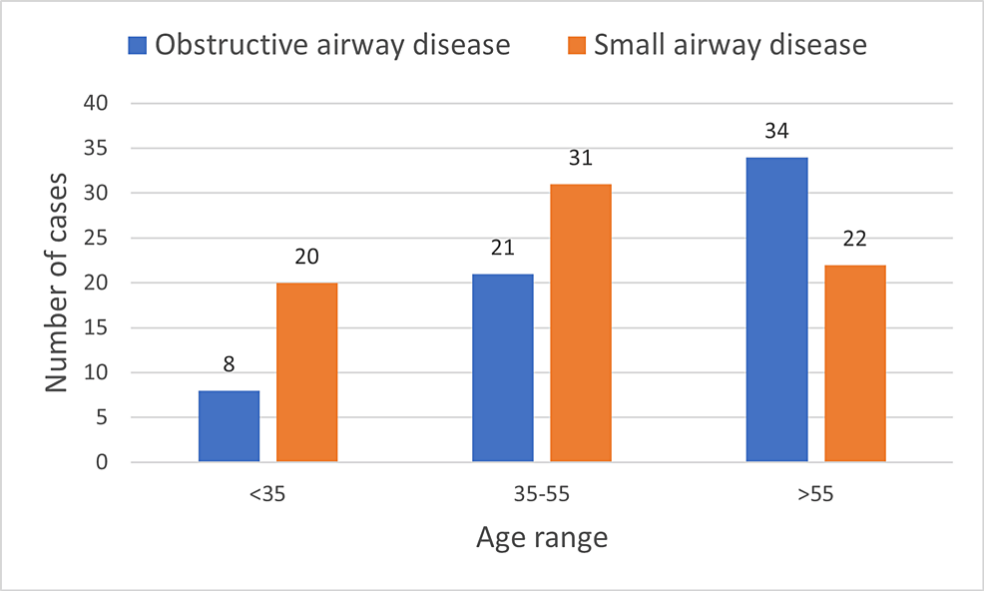
Distribution of obstructive pattern of spirometry and small airway disease among age groups.

The distribution of airflow obstruction by age was with eight subjects in those ≤35 years of age (5.4%), 21 subjects in those between 36-55 years (7.8%), and 34 in those >55 years (25%) (Figure 3). Thirty-five subjects provided a history of smoking. The average pack years was 9.7 pack years. Eighteen smokers were in the 35-55 age group with an average of seven pack years. Ten smokers were in the age group above 55 with an average pack year of 19.8, whereas the seven smokers in the 18-35 age group had an average of two pack years. Only nine smokers had abnormal spirometry with obstructive pattern (OAD or small airway disease).

Table 3 shows the distribution of obstructive airway disease and small airway disease across different age ranges, BMI groups, smoking history, inhaler use, and family history. Thirty-nine percent of those who had obstructive airway disease had respiratory symptoms like chronic cough, dyspnea, wheezing, etc., while 23% of small airway diseases were symptomatic. Of all the parameters, age, family history of asthma, respiratory symptoms, and inhaler use were found to have a statistically significant association with obstructive airway disease.

**Table 3:** Characteristics of population with obstructive defect and small airway disease on spirometry with statistical significance.

Defect type			
	Obstructive (N=63)	Small airway disease (N=73)	P value
Age			0.00001
<35 years	8 (12.7%)	20 (27.4%)	
35-55 years	21 (33.4%)	31(42.4%)	
>55 years	34 (53.9%)	22 (30.2%)	
Age mean(SD)	46.52 (13.6%)	45.76(13.8%)	0.0006
BMI			0.8446
<18	3 (4.7%)	3 (4.1%)	
18-25	18 (28.5%)	25 (34.3%)	
25-30	36 (57.3%)	30 (41.1%)	
>=30	6 (9.5%)	15 (20.5%)	
BMI mean (SD)	26.1 (5.2%)	26.6 (4.9%)	0.8199
Sex			0.9051
male	36 (67.8%)	47 (64.4%)	
female	27 (32.2%)	26 (35.62%)	
Inhaler use			0.002351
Yes	19 (30.1%)	7 (9.6%)	
No	44 (69.9%)	66 (90.4%)	
Asthma family history			0.0092
Present	14 (22.3%)	5 (6.8%)	
Absent	49 (77.7%)	68 (93.2%)	
Smoking history			0.4186
Yes	3 (4.8%)	6 (8.2%)	
No	60 (95.3%)	67 (91.8%)	
Respiratory symptoms			<0.001
Yes	25 (39.6%)	17 (23.2%)	
No	38 (60.3%)	56 (76.7%)	

To summarize, of the total 538 subjects incorporated in the study, the overall yield from spirometry in detecting airflow obstruction was 63 subjects (11.7%). Sixteen subjects (25.4%) had a mild degree of obstructive defect, 36 (57.2%) subjects had a moderate obstructive defect, 10 (15.9%) had a severe obstructive defect, and only one (1.5%) spirometry demonstrated a very severe obstructive defect. Fifty-five subjects (10.2%) were in the restrictive spirometry group. Seventy-three subjects (13.5%) were classified as small airway disease. The detection rates of obstructive airway disease and small airway disease were similar in both males and females. The average pack years was 9.7 pack years. Although overall smoking history showed no significant association with developing airflow obstruction, significant association with smoking was found in the elderly (>55) age group.

## Discussion

This study sought to investigate the efficacy of spirometry in determining the prevalence of airflow obstruction as per FEV_1_/FVC ratio ≤70% in a routine health checkup population in Mumbai, India. This study also sought to determine whether spirometry as a screening test should be used in a general population or specific groups with certain risk factors like age, BMI, gender, family history of asthma, and smoking. As mentioned earlier, individuals with previously spirometric-diagnosed restrictive or obstructive airway diseases were excluded from the study so that they do not influence the results of these studies.

The prevalence of airflow obstruction in this study was 11.7%. A study by Chakrabarti et al. [6] in West Bengal analyzed the spirometry of 286 subjects, and 16% were noted to exhibit airflow obstruction, and the factors associated were increasing age, smoking history, male gender, reduced BMI, and occupation. In the Nippon COPD Epidemiology Study [7], the prevalence of airflow limitation was reported in 10.9% of 2343 subjects aged >40 years on clinical and spirometric indices. The prevalence of airflow obstruction in the study was 5.7% in a study on spirometric data by Manzar et al. [8] in people undergoing a health checkup in Karachi, Pakistan. In another study conducted by Xu et al. [5] in China, the detection rate of COPD and obstructive small airway disease was 0.8% and 4.0%, respectively, showing a positive correlation with the male gender, age, and smoking index. Another community-based project Takemura et al. [9] observed a prevalence of 3.6% in all subjects of Japanese origin.

Compared to these studies, the detection rates of both obstructive pattern and small airway disease in this study are higher. However, a comparison between this study and other studies is likely to be biased owing to the different study populations and different criteria used for assessing airflow limitation. Our study used the GOLD criteria for diagnosis of airflow obstruction (FEV_1_/FVC<70%).

Our study demonstrated increasing age associated with airflow obstruction. Almost 25% of study subjects in the elderly age group (>55 years) showed an obstructive pattern of spirometry. Similar associations between age and airflow obstruction have been described in studies worldwide [8-10]. Aging is a risk factor for COPD, and the incidence of COPD increases along with age [11]. Thus, special attention should be paid to the screening of obstructive airway diseases in middle-aged and elderly populations. This also emphasizes the role of environmental factors, urbanization, and industrialization, which play a role in the deterioration of lung function as a person ages, in association with other risk factors.

An epidemiological study in China indicated that low BMI might increase the risk of COPD, especially in the elderly (60 years and over) populations [12]. However, in our study, BMI was not significantly associated with airflow obstruction. Hence, the relationship between BMI and COPD still requires further verification in more epidemiological studies with larger sample sizes.

Male sex and increasing age are identified as risk factors in most studies from Asia [13-15]. This relationship may also be attributed to the greater prevalence of smoking among men and the cumulative effects of smoking and other exposures with age. However, more recent data from developing countries have reported that the prevalence of COPD is now equal in males and females, reflecting the changing trends of tobacco smoking [13]. In our study, the detection rates of obstructive airway disease were similar in both males and females and did not establish any significant association between gender and airflow obstruction.

Smokers constituted 6.5% of subjects, with an average of 9.7 pack years. The average pack years in the elderly age group was 19.8. However, there is a possibility that some subjects may have confounded their smoking history. In elderly subjects, smoking was found to be significantly associated with airflow obstruction. However, in the young and middle-aged groups, smoking history did not attain any statistical significance. This is likely because obstructive airway diseases like COPD predominantly require a cumulative smoking history of approximately 20 pack years or more. It is very unlikely for an individual aged less than 35 years to accumulate a 20-pack-year smoking history. This supports the fact that the duration of tobacco smoking and the number of cigarettes smoked have a cumulative effect on developing airflow obstruction. Therefore, as a global joint effort, effective measures should be taken to lower the smoking rate, eliminate the hazards of tobacco, and maintain and promote human health.

In our study, family history was found to be significantly associated with the occurrence of airflow obstruction. Other studies have also identified that family history of asthma in one or more first-degree relatives as a risk factor for asthma [16]. Asthma symptoms and signs are also variable in nature and can demonstrate normal or abnormal spirometry depending on the day it is performed.

Dyspnea and cough were the most common symptoms reported by the subjects diagnosed with airflow obstruction (11%). Airflow obstruction was found to be associated with respiratory symptoms like dyspnea, cough, and wheezing. Medbo et al. [17] emphasized that respiratory symptoms are valuable predictors of airflow limitation and the presence of two or more symptoms should prompt the physician to investigate the patient for the presence of airflow limitation.

The study was conducted in Mumbai, a major urban city with significant air pollution because of vehicular emissions, commercial, and industrial activities. This can lead to both short-term, acute, and long-term chronic respiratory problems. The long-term effects of this air pollution can cumulatively lead to chronic irritation of the airways, which initially can cause small airway disease and eventually also lead to chronic obstructive pulmonary disease. These effects happen over time and may demonstrate a spirometry-measurable disease only after significant cumulative years of exposure. This likely contributes to the increasing incidence of obstructive airway diseases in the population above 35 years of age, irrespective of smoking. As we know, the burden of air pollution in India is ever-increasing, and, therefore, the findings of the study can be extrapolated to such age groups across India. This study demonstrates that 25% of the individuals above 55 years of age had obstructive spirometry. This is significant and therefore advocates for screening spirometry in this age group. We will include this clarification in the study. In our current study, the incidence of small airway disease reached 13.5%. Small airway disease may progress to COPD if the modifiable risk factors are not interrupted timely. Therefore, timely identification of these individuals and early intervention are particularly valuable.

In this study, 55 individuals (10%) were found to have restrictive airway disease. Restrictive lung diseases may be present in various conditions ranging from primary pulmonary causes like lung parenchymal diseases, interstitial lung disease, pneumoconiosis, sarcoidosis, etc. It can also result because of various extrathoracic causes like obesity, kyphoscoliosis, neuromuscular diseases affecting the diaphragm, ascites, etc. This list of causes of restrictive lung disease is extensive and can be multifactorial. This needs a separate dedicated restrictive lung disease screening which would include chest X-ray, blood tests, CT chest imaging, advanced spirometric studies like diffusion testing, lung volumes, and workup for extrathoracic causes.

Limitations

In this study, there was no statistically significant association found between smoking and obstructive airway disease in the young and middle-aged groups. However, it is possible that some individuals may have confounded their smoking history. Therefore, the association between smoking and obstructive airway disease in this study may be underestimated. Most of the study subjects belonged to the young or the middle-aged age groups. The health checkup population are often composed of both healthy people and people with well-controlled chronic diseases like diabetes, hypertension, ischemic heart disease, hypothyroidism, chronic kidney disease, etc. In comparison to other studies, our study was conducted in the urban population and, hence, had good economic status and lower chances of being exposed to biomass fuel and childhood respiratory infections. In view of all these factors, it is difficult to extrapolate the results of this study to the general Indian population. Therefore, we recommend the need for similar spirometric studies with larger population groups across different parts of the country. Although this study sought to estimate the prevalence of both obstructive and restrictive airway disease, the focus of this study was more on obstructive airway disease. This is because obstructive airway disease is largely because of pulmonary causes whereas restrictive airway diseases may be because of pulmonary and extrapulmonary causes. Therefore, restrictive airway defects found on spirometry may trigger further systemic laboratory and radiological workup based on the history and symptoms. Hence, we recommend the need for a separate restrictive airway disease study to analyze the factors influencing restrictive airway defect and to characterize the target population who should receive screening spirometry.

## Conclusions

In conclusion, increasing age, respiratory symptoms, smoking history in the elderly, and family history of asthma are factors associated with airflow obstruction. There was no association between gender and airflow obstruction. Also, the association between BMI and airflow obstruction was not significant in this study. This study advocates the use of screening spirometry in subjects of both sexes above 35 years of age with or without any smoking history, people with family history of asthma, people with respiratory symptoms, and elderly people with moderate smoking history for early detection of airflow obstruction in routine health checkup population.

## Appendices

Study Questionnaire

Code No.: ___________                DOB: _______________       Gender: ____________

Age:

Respiratory complaints: Breathlessness/chronic cough/frequent nose block/frequent sneezing 

Smoking: YES/NO/QUIT

                  If YES _____cigarettes/day for ____ yrs 

Profession:

Past medical history:

Inhaler use:

Family history of asthma:

Spirometry test result:

Conclusion

_________________________                                            ______________________

       Guide’s Signature                                                             Investigator’s Signature 

Informed Consent Form 

Participant’s Initials: _________________ Participant’s Name:    ___________________ 

Date of Birth / Age:  _________________

          Please Initial Box                                                                                            (Participant) 

(i) I confirm that I have read and understood the information sheet dated                     -----------------          

 for the above study and have had the opportunity to ask questions. 

(ii) I understand that my participation in the study is voluntary and that I am free  

free to withdraw at any time, without giving any reason, without my medical              -----------------

care or legal rights being affected. 

(iii) I understand that the study investigator and study team, the Ethics                    

Committee and the regulatory authorities will not need my permission to look 

 at my health records both in respect of the current study and any further                  -----------------

research that may be conducted in relation to it, even if I withdraw from the study.   

I agree to this access.  However, I understand my identity will not be revealed in any information which may get published. 

(iv) I agree not to restrict the use of any data or results that arise from this study     -----------------

provided such a use is only for scientific purpose(s). 

(v) I agree to take part in the above study.                                                                 -----------------

---------------------------               --------------------                                        ----------------- 

Name of the Participant         Sign/Thumb Impression                                        Date

-----------------------------            -------------------                                          -----------------

Name of LAR                       Sign/Thumb Impression                                        Date

(Legally Acceptable Representative)

---------------------------             --------------------                                         -----------------

Name of the Investigator                 Sign                                                            Date

-----------------------------             -------------------                                          -----------------

Name of Impartial Witness              Sign                                                           Date
